# Beyond the Norm: Atypical Manifestations of Aspergillosis

**DOI:** 10.1002/rcr2.70525

**Published:** 2026-03-01

**Authors:** Leila Zahiri, Mohammad Javad Fallahi, Massood Hosseinzadeh, Nazanin Alemzadeh, Zahra Ghanbarinasab, Fatemeh Razmjooei

**Affiliations:** ^1^ Pulmonary Research Center Shiraz University of Medical Science Shiraz Iran; ^2^ Thoracic and Vascular Surgery Research Center Shiraz Iran; ^3^ Department of Pathology Shiraz University of Medical Science Shiraz Iran; ^4^ Hematology Research Center, Department of Hematology, Medical Oncology and Stem Cell Transplantation Shiraz University of Medical Sciences Shiraz Iran; ^5^ Student Research Committee Jahrom University of Medical Science Jahrom Iran

**Keywords:** aspergillus infection, chest wall mass, immunocompromised patient

## Abstract

Aspergillus fumigatus is a significant pathogen responsible for a wide spectrum of pulmonary diseases, ranging from allergic reactions to invasive pulmonary aspergillosis, particularly among immunocompromised individuals. We describe a 62‐year‐old female kidney transplant recipient who presented with chest pain, a right anterior chest wall mass, cough and anorexia of 2 months' duration. Laboratory evaluation revealed elevated inflammatory markers, prompting consideration of malignancy and infection. Bronchoalveolar lavage was positive for Aspergillus galactomannan antigen, and biopsy of the chest wall mass confirmed Aspergillus infection. This case represents an unusual manifestation of Aspergillus involving soft tissue. Accurate diagnosis requires histopathological confirmation and molecular testing. This report emphasizes the importance of including fungal infections, particularly Aspergillus species, in the differential diagnosis of soft tissue masses in immunocompromised patients.

## Graphical abstract

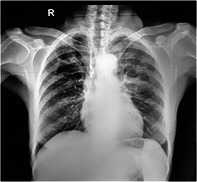

## 1 Introduction

Aspergillus fumigatus is a ubiquitous saprotrophic fungus and a major opportunistic pathogen implicated in a broad spectrum of pulmonary diseases. Despite the availability of antifungal therapies, it remains a leading cause of severe lung pathology worldwide, contributing to substantial morbidity and mortality. Transmission occurs primarily through inhalation of airborne conidia, which are detected not only in patients with pulmonary disease but also in asymptomatic individuals; notably, fungal DNA has been reported in up to 37% of lung biopsies from otherwise healthy adults [1, 2].

The clinical spectrum of aspergillosis is remarkably diverse and strongly influenced by the host's immune status. While allergic syndromes such as asthma and hypersensitivity reactions represent milder forms, more severe manifestations include aspergilloma and invasive pulmonary aspergillosis (IPA) [3]. Diagnosis requires an integrated approach, combining clinical presentation, imaging and laboratory studies. Typical features may include productive cough, dyspnea, weight loss and hemoptysis, while radiological findings and serological tests provide essential diagnostic support.

In recent decades, the expanding population of immunocompromised patients has significantly increased the burden of invasive fungal infections. IPA has emerged as the most common invasive fungal disease among solid organ and haematopoietic stem cell transplant recipients [4]. Moreover, immunosuppressed hosts often present with atypical and misleading clinical features; for instance, invasive tracheal aspergillosis has been documented following chemotherapy [5].

Here, we describe a rare case of Aspergillus infection presenting as a chest mass, underscoring the diagnostic challenges of fungal diseases in immunocompromised patients and highlighting the importance of considering unusual manifestations in clinical practice.

## 2 Case Report

A 62‐year‐old woman with a history of kidney transplantation 9 months prior to presentation was admitted with a two‐month history of right‐sided chest pain radiating to the right scapula and axilla. Over the 3 weeks preceding admission, she developed a tender, palpable mass in the right upper anterior chest wall, associated with overlying erythema, cough, anorexia and an unintentional weight loss of 6 kg over 2 months. She denied fever or night sweats.

The patient was a retired secretary with no known occupational or environmental exposure to fungal pathogens. Her past medical history was significant for urinary tuberculosis treated 40 years earlier, which required surgical intervention resulting in left nephrectomy, cystectomy and partial resection of the right kidney. There was no history of diabetes mellitus, recent infections or malignancy. HIV serology was negative. Information regarding donor type was unavailable.

At the time of presentation, the patient was receiving maintenance immunosuppressive therapy consisting of prednisolone 7.5 mg once daily, tacrolimus 3 mg orally once daily, sirolimus 2 mg orally once daily and azathioprine 50 mg orally once daily. Additional medications included diltiazem 60 mg once daily and pantoprazole 40 mg once daily. She was not receiving antimicrobial prophylaxis, as routine post‐transplant prophylaxis had been discontinued after the first 6 months in accordance with standard practice.

Physical examination revealed a tender, palpable mass over the right anterior chest wall with overlying erythema, along with crackles localized to the right upper lung lobe. No other significant abnormalities were noted.

Initial laboratory evaluation demonstrated leukopenia, with a white blood cell count of 1.8 × 10^3^/μL. The differential leukocyte count showed polymorphonuclear cells 89.3% and lymphocytes 7%. Baseline leukocyte counts prior to presentation were unavailable. Inflammatory markers were markedly elevated, including ferritin 1163 ng/mL, erythrocyte sedimentation rate (ESR) 104 mm/h and C‐reactive protein (CRP) 32 mg/L, raising concern for an infectious or malignant process.

Given the leukopenia, a peripheral blood smear (PBS) and bone marrow aspiration and biopsy were performed to evaluate for hematologic malignancy. The PBS revealed mild leukopenia with polymorphonuclear toxic granulation, absence of cytoplasmic vacuolization and fragmented red blood cells comprising less than 1% of cells. Bone marrow biopsy demonstrated mild hypocellularity without evidence of malignant infiltration or metastatic disease. The leukopenia was considered most likely related to azathioprine therapy; following discontinuation of azathioprine, the patient's white blood cell count improved, supporting a drug‐related aetiology.

To assess potential infectious sources and graft status, an abdominopelvic ultrasound was performed. Sonography demonstrated the absence of both native kidneys, consistent with prior surgical history. The transplanted kidney measured 107 × 43 × 56 mm, exhibited normal parenchymal echogenicity, and showed no evidence of hydronephrosis, nephrolithiasis, or perinephric fluid collection.

**FIGURE 1:**
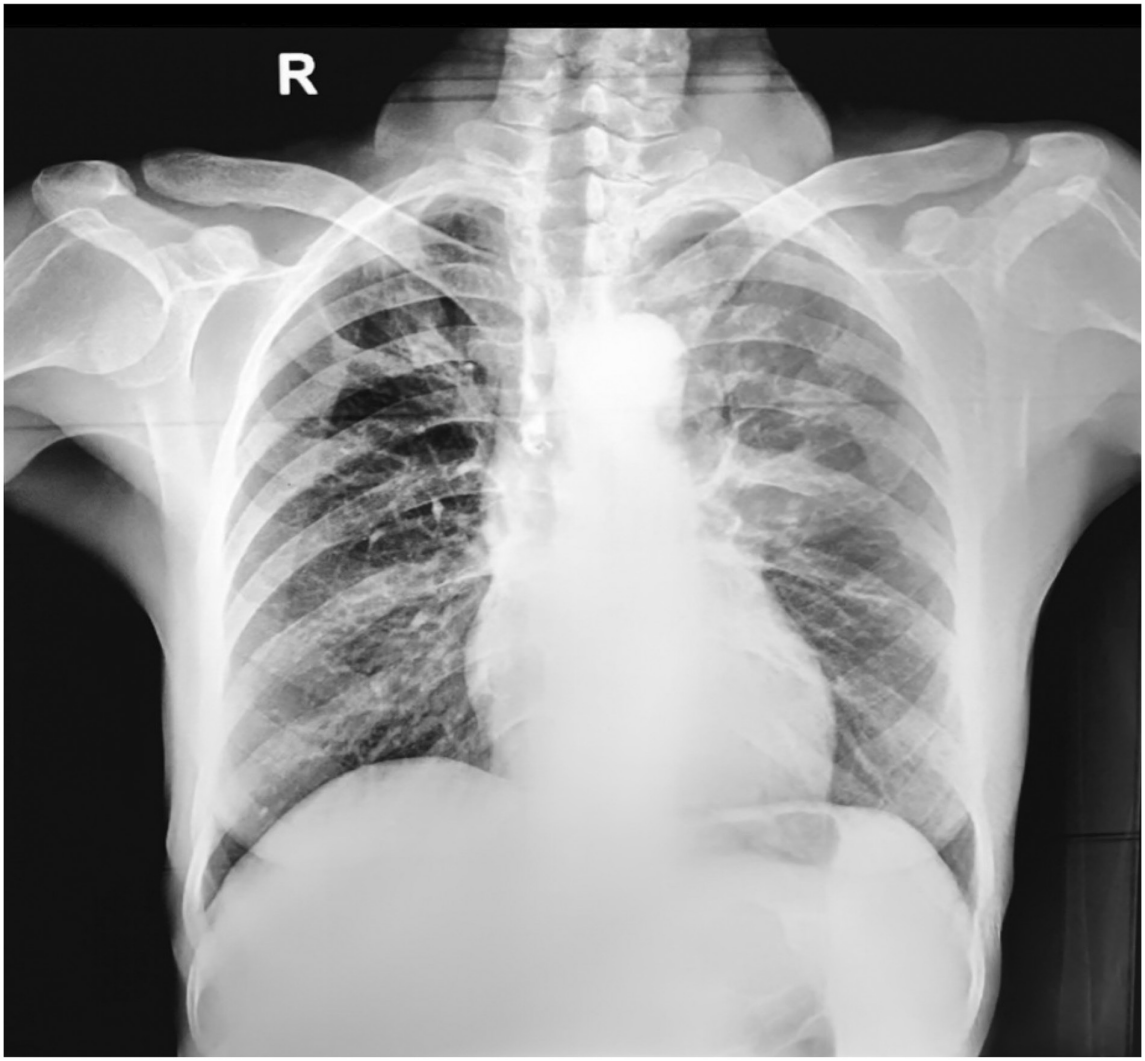
Chest X‐ray.

To further evaluate the chest wall mass, soft tissue ultrasonography was performed, revealing a hypoechoic, heterogeneous lesion measuring 21 × 23 × 17 mm (approximately 4 cc), located medially in the right upper anterior chest wall adjacent to the third and fourth ribs, with posterior extension suggestive of a collection. Chest radiography demonstrated hyperlucency and air trapping in the right upper lobe (Figure 1).

**FIGURE 2:**
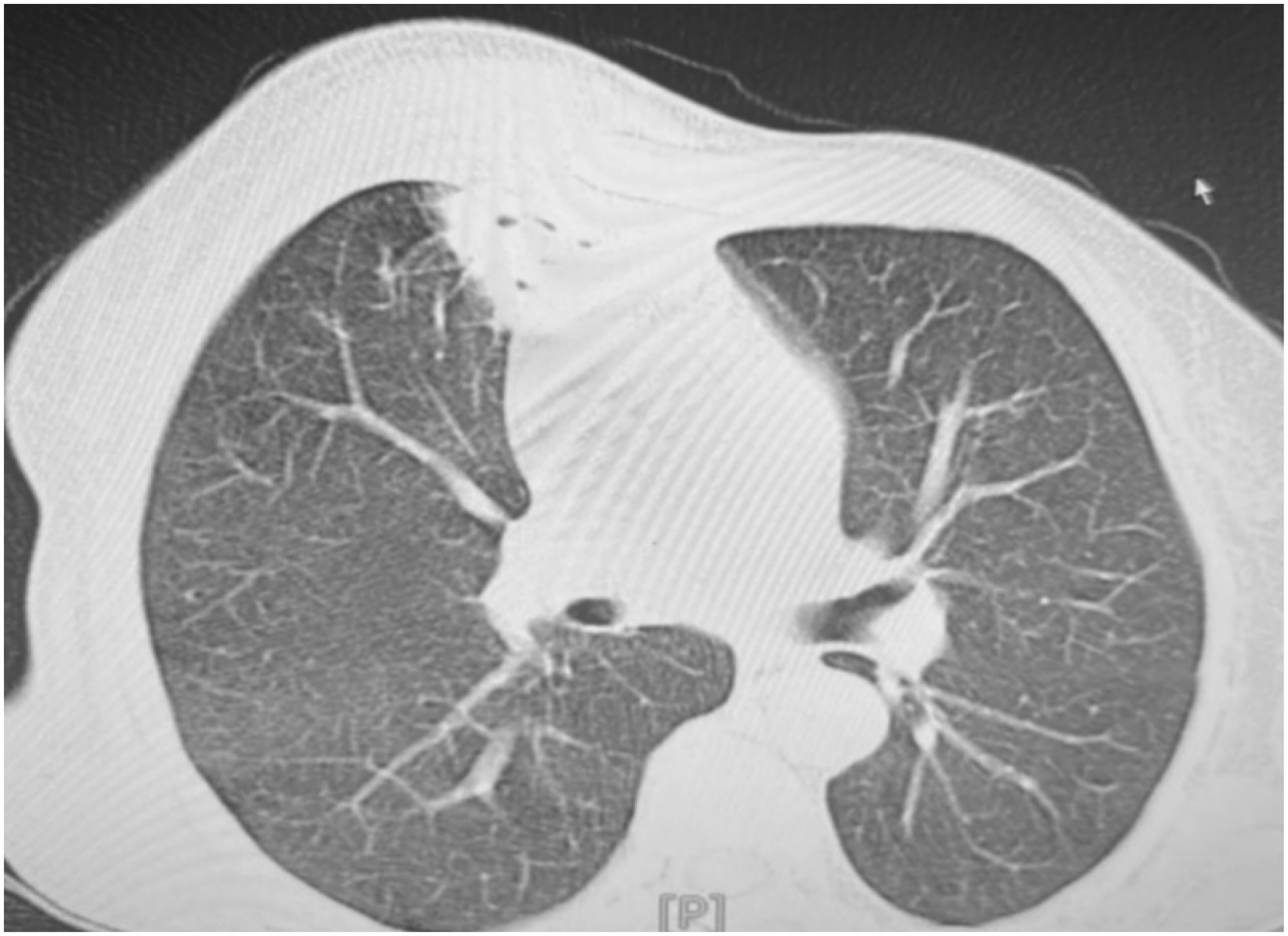
CT showing lung mass.

Chest computed tomography (CT) showed a bilateral pleural effusion. In addition, a cavitary lesion measuring approximately 44 × 50 mm was identified in the left upper lobe, along with a second cavitary lesion measuring approximately 31 × 17 mm in the anterior segment of the right upper lobe (Figure 2). No atoll (reverse halo) sign was observed.

Bronchoscopy was performed to assess pulmonary involvement. No endobronchial abnormalities were identified. Bronchoalveolar lavage (BAL) samples obtained from both lungs were negative for gram stain, bacterial cultures and acid‐fast bacilli. However, BAL Aspergillus galactomannan testing was positive, with a value of 2.77 (positive threshold > 0.5). Aspergillus PCR could not be performed on the BAL sample due to limited sample availability. Instead, PCR testing for aspergillosis was performed on peripheral blood and the chest wall mass, both of which yielded positive results. Transbronchial lung biopsy was not performed because of the absence of endobronchial lesions and concerns regarding procedural risk in an immunosuppressed patient.

**FIGURE 3:**
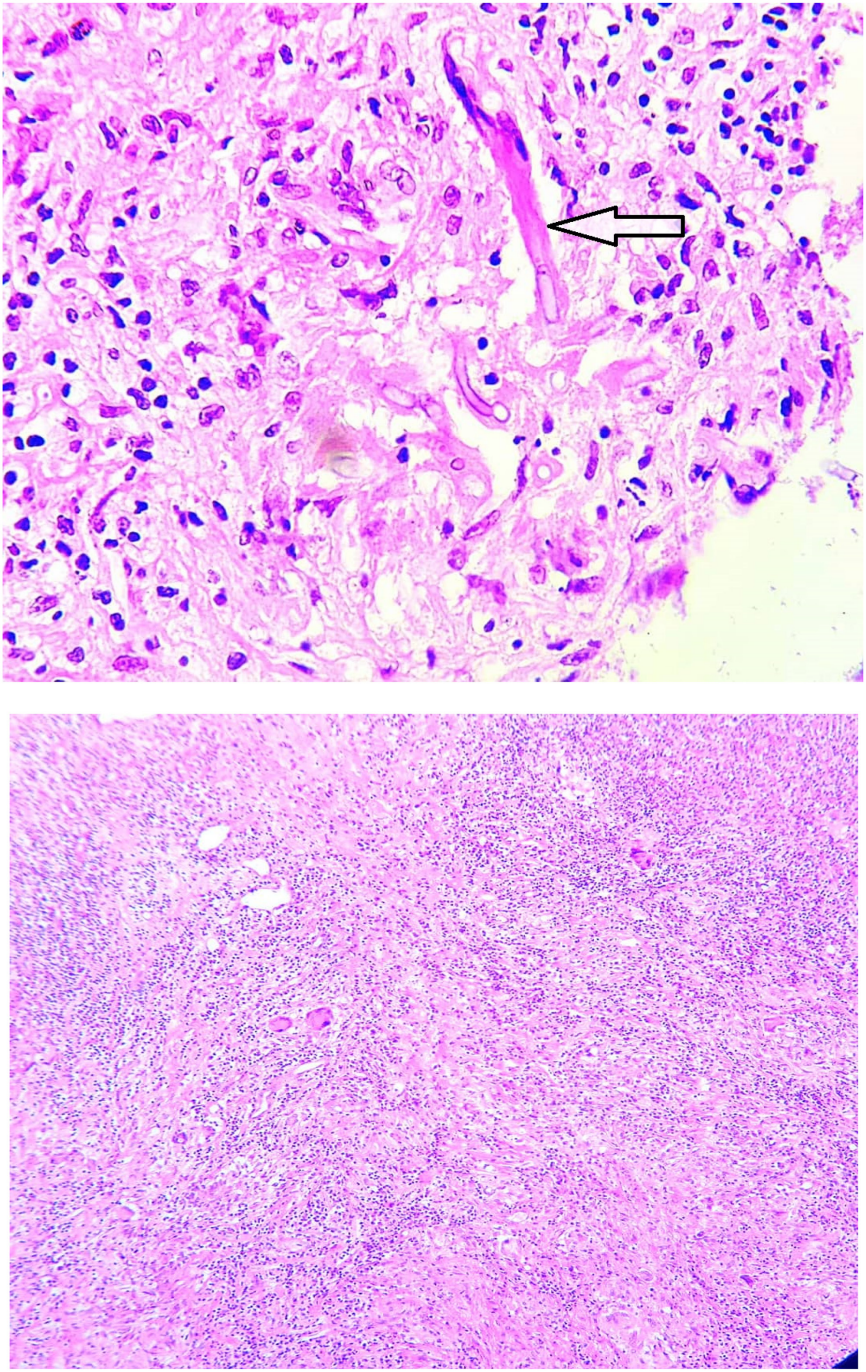
Histopathological evaluation of the chest wall mass.

A biopsy of the chest wall mass was subsequently performed. Histopathological examination revealed focal necrosis with acute and chronic inflammatory infiltrates and fungal elements were identified. No evidence of malignancy was observed in the fully embedded specimen, and no lung tissue was present (Figure 3).

Polymerase chain reaction analysis of the biopsy specimen was negative for BK virus, cytomegalovirus and mucormycosis, but confirmed the presence of Aspergillus DNA. In addition, Aspergillus PCR performed on peripheral blood was positive.

Serum creatinine at admission was 1.43 mg/dL, which increased to a peak of 1.61 mg/dL during hospitalization. Following initiation of antifungal therapy, renal function gradually normalized, and the patient was discharged with a serum creatinine level of 1.06 mg/dL. Graft function remained stable throughout the clinical course, with no evidence of graft rejection.

**FIGURE 4:**
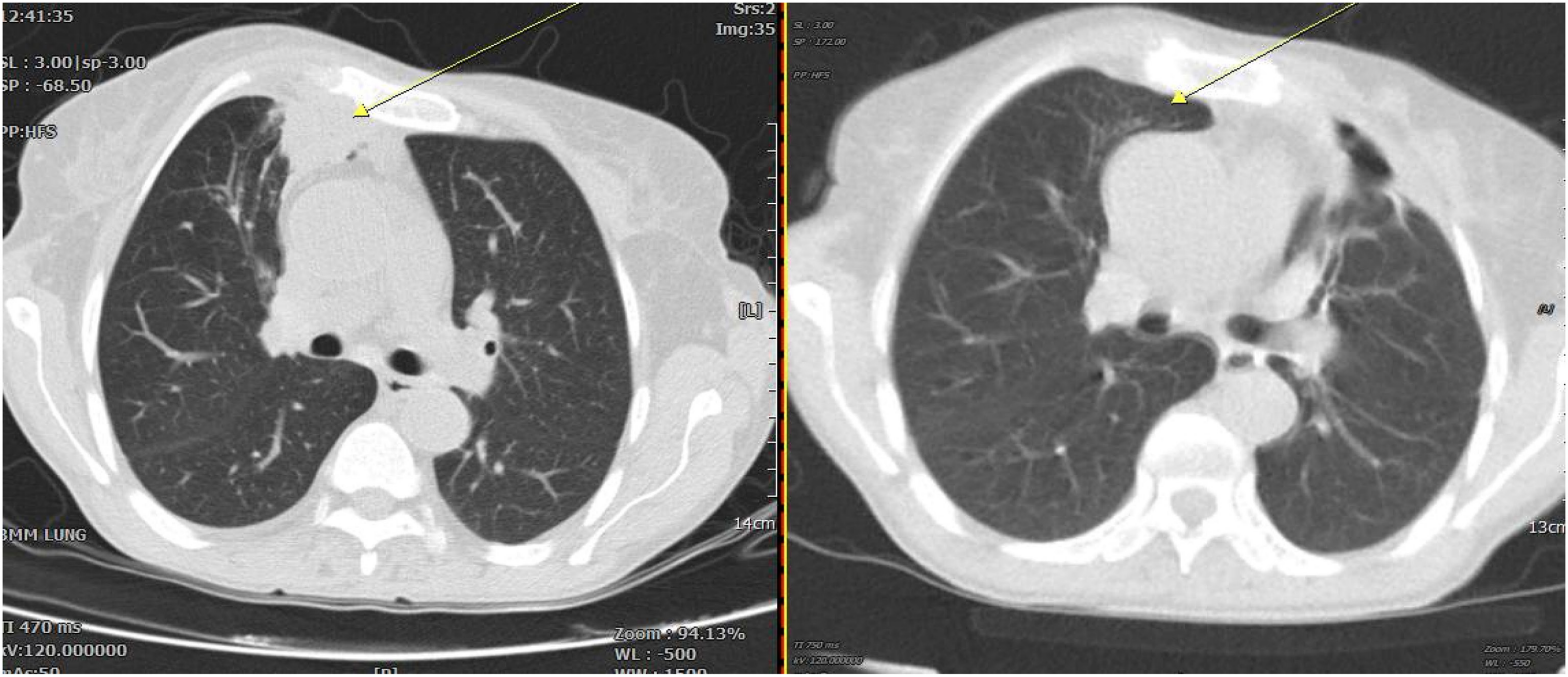
Comparative chest CT scans demonstrating findings at presentation (left) and follow‐up after 6 months of antifungal therapy (right).

Based on the clinical presentation, immunosuppressed status, radiologic findings and microbiological and histopathological evidence, a diagnosis of atypical invasive aspergillosis was established. The patient was treated with voriconazole, which was initiated with an intravenous loading dose of 300 mg, followed by 300 mg every 12 h for two doses. Maintenance therapy was then continued at 200 mg intravenously every 12 h. After 4 days of treatment, the patient showed clear clinical improvement, and therapy was transitioned to oral voriconazole at a dose of 200 mg every 12 h, which was continued thereafter. It should be mentioned that due to known drug–drug interactions between voriconazole and immunosuppressive agents, close monitoring and dose adjustments were undertaken. During follow‐up, the dose of sirolimus was reduced. Tacrolimus levels were monitored regularly at three‐month intervals and remained within the therapeutic range without exceeding toxic levels. In addition, diltiazem was gradually discontinued and replaced with valsartan to minimize potential pharmacokinetic interactions. Prednisolone was continued at a maintenance dose of 7.5 mg daily. These measures allowed for safe administration of voriconazole without adverse effects related to immunosuppressive therapy. After 6 months of antifungal therapy, follow‐up imaging demonstrated near‐complete resolution of the chest wall mass. Comparative chest CT scans obtained at presentation and after 6 months of treatment showed marked regression of the lesion (Figure 4). She is now clinically stable under follow‐up.

## 3 Discussion

Soft tissue aspergilloma is a rare form of fungal infection caused by Aspergillus species. Immunocompromised individuals, as well as patients with uncontrolled diabetes, haematological malignancies and rheumatologic conditions, are at higher risk of developing this infection [6, 7, 8].

The antigenicity of Aspergillus is primarily attributed to β‐glucans and its enzymatic activity, which induce an inflammatory response and contribute to tissue necrosis. Hematogenous dissemination may facilitate the migration of Aspergillus to soft tissues [9].

Soft tissue aspergilloma can occur secondary to invasive pulmonary aspergillosis. For instance, Fan et al. reported a case of a 43‐year‐old male patient who presented with invasive pulmonary aspergillosis with extension into the maxillofacial soft tissues. This patient had agranulocytosis induced by methimazole, which he had been taking for hyperthyroidism [7]. Similarly, Mahendra et al. described a 55‐year‐old male who presented with a soft tissue mass on the right side of his nose, initially misdiagnosed as a tuberculoma. Soft tissue aspergilloma is an uncommon entity that is frequently misdiagnosed as a neoplastic or inflammatory lesion [2, 10].

Our case describes a 62‐year‐old female patient who initially presented with localized chest pain, which progressed to a soft tissue mass within 3 weeks. In this case, the absence of significant immunosuppression raises the possibility of direct inoculation or a chronic low‐grade infection, as evidenced by a positive Aspergillus result in a bronchoalveolar lavage (BAL) specimen.

Histopathologic investigations played a crucial role in establishing the diagnosis, revealing septate hyphae with branching, characteristic of Aspergillus [11]. PCR confirmed the fungal aetiology, emphasizing the importance of molecular techniques in diagnosing deep‐seated fungal infections.

The treatment of soft tissue aspergilloma varies depending on the extent of disease and individual patient factors. Antifungal therapy with triazoles, such as voriconazole, remains the primary treatment due to its superior efficacy against Aspergillus species [12]. In cases where the lesion exerts a mass effect, surgical intervention may be necessary.

The importance of considering fungal infections in the differential diagnosis of soft tissue masses is underscored, particularly in cases involving chronic or atypical lesions. A multidisciplinary approach, incorporating microbiological, histopathologic and molecular diagnostics, is essential for accurate diagnosis and effective management.

## Author Contributions

All authors contributed to the collection of data, manuscript development and final approval. **Leila Zahiri:** methodology, conceptualization, project administration, resources, supervision, validation. **Zahra Ghanbarinasab:** data curation, investigation, project administration, writing, original draft. **Nazanin Alemzadeh:** data curation, investigation, writing, original draft, writing, review and editing. **Fatemeh Razmjooei:** data curation, investigation, writing, original draft. All authors have read and agreed to the published version of the manuscript.

## Funding

The authors have nothing to report.

## Disclosure

Declaration of Generative AI and AI‐Assisted Technologies in the Writing Process: During the preparation of this work the authors used ChatGPT in order to improve language and readability. After using this tool, the authors reviewed and edited the content as needed and take full responsibility for the content of the publication.

## Ethics Statement

In accordance with international ethical standards, the authors have obtained and securely retained written ethical permission.

## Consent

The authors declare that written informed consent was obtained for the publication of this manuscript and accompanying images using the form provided by the Journal.

## Conflicts of Interest

The authors declare no conflicts of interest.

## Data Availability

The data that support the findings of this study are available from the corresponding author upon reasonable request.
